# Molecular docking and simulation analysis of c-KIT and PDGFRα with phytochemicals as dual inhibitors for GIST

**DOI:** 10.6026/973206300200974

**Published:** 2024-09-30

**Authors:** Kaoutar El Khattabi, Sanaa Lemriss, Rachid El Jaoudi, Fouad Zouaidia

**Affiliations:** 1 Medical Biotechnology Laboratory, Rabat Medical and Pharmacy School, Mohammed V University in Rabat, Rabat, Morocco; 2 Department of Biosecurity PCL3, Laboratory of Research and Medical Analysis of the Fraternal of Gendarmerie Royale, Rabat, Morocco; 3 Pathology Department, Ibn Sina University Hospital, Rabat, Morocco

**Keywords:** GIST, multi-drug target, c-KIT, PDGFRα, molecular docking, molecular dynamics

## Abstract

Mutations in the c-KIT or PDGFRα genes primarily drive gastrointestinal stromal tumors (GISTs). While tyrosine kinase inhibitors (TKIs) such as Imatinib have improved outcomes, resistance due to secondary mutations remains a significant challenge. This study used computational methods to identify phytochemicals from Moroccan plants as dual inhibitors of c-KIT and PDGFRα. Screening 545 phytochemicals, 6-Hydroxygenistein (6-OHG), a derivative of Genistein, showed high binding affinities (-10.3 kcal/mol for PDGFRα and -10.5 kcal/mol for c-KIT), comparable to Imatinib. 6-OHG demonstrated competitive binding affinities, favorable ADMET properties, good solubility, and oral bioavailability. Its antioxidant properties suggest a potentially lower toxicity profile. Interaction analysis revealed significant hydrogen bonds and hydrophobic interactions with key residues in both targets. Molecular dynamics simulations over 30 ns indicated stable complexes with consistent RMSD values, radius of gyration, solvent-accessible surface area, and hydrogen bonding patterns. Free binding energy calculations using the MM-PBSA method highlighted strong binding efficacy, with total binding energies of -278.0kcal/mol for PDGFRα and -202.1kcal/mol for c-KIT, surpassing Imatinib. These findings suggest that 6-OHG is a promising dual inhibitor for GIST therapy, potentially overcoming resistance mechanisms associated with current TKIs. However, further experimental validation is necessary to fully understand it's potential.

## Background:

Gastrointestinal stromal tumors (GISTs) are the predominant mesenchymal tumors within the gastrointestinal tract, primarily driven by mutations in the c-KIT or PDGFRα genes [1]. Introducing tyrosine kinase inhibitors (TKIs), such as imatinib, has dramatically improved patient therapeutic outcomes. However, imatinib resistance often emerges due to secondary mutations in the kinase domains of these genes, particularly KIT and PDGFRA, which reduce the drug's effectiveness over time [2]. This issue has spurred urgent research toward developing novel inhibitors or combination therapies capable of overcoming such resistance [3]. Though available, second-line agents like Sunitinib and Regorafenib also encounter resistance and adverse effects [4]. Phytochemicals, bioactive compounds derived from plants, have shown significant potential in cancer therapy by modulating multiple signaling pathways with minimal toxicity [5]. For example, compounds such as resveratrol have been shown to inhibit kinases, including PDGFRα, suggesting their therapeutic relevance in targeting these pathways [6]. Targeting both c-KIT and PDGFRα offers therapeutic advantages by circumventing resistance mechanisms from mutations in either gene, leading to a more comprehensive treatment approach [7]. Computational studies indicate that specific structural characteristics can enhance the inhibition of both kinases, potentially resulting in more effective therapies with reduced off-target effects [8]. The pressing need for novel inhibitors or combination therapies in GIST treatment drives this research. Therefore, it is of interest to use molecular docking and dynamics simulations to identify and validate Moroccan phytochemicals as dual inhibitors of c-KIT and PDGFRα.

## Materials and Methods:

## Protein preparation:

The crystal structures of PDGFRα (PDB ID: 6JOL) and c-KIT (PDB ID: 1T46) were obtained from the Protein Data Bank (PDB) with a resolution of 1.90 Å. These sequences were then prepared for docking using PyMOL software, and we modeled all missing residues using the Swiss Model server [9].

## Definition of the active site and functional residues:

The active and binding sites for PDGFRα and c-KIT were identified using UniProt data. For PDGFRα, the ATP binding site residues are 599-607 and 627, with the proton binding site at residue 818. For c-KIT, the ATP binding site residues are 596-603, 623, 671-677, and 776, and the proton binding site is at residue 792 [10].

## Phytochemicals library preparation:

The Moroccan Phytochemicals Database (MPDB) was used to export three-dimensional structures of 545 phytochemicals from Moroccan aromatic and medicinal plants [11].

## Virtual screening:

Docking simulations were conducted and validated using the re-docking method using PyRx software. Compounds were selected based on binding affinity and Root-Mean-Square Deviation (RMSD) values [12].

## Ligand-Receptor interaction analysis:

Interaction analysis between ligands and receptors was visualized using Discovery Studio Visualizer, focusing on hydrogen bonding and hydrophobic interactions [13].

## Drug-Like properties of phytochemicals:

The drug-like properties of top-docked phytochemicals were evaluated: absorption, distribution, metabolism, excretion, and toxicity (ADMET) profiles using FafDrug4 [14].

## Molecular dynamics simulation:

Molecular Dynamics (MD) simulations using GROMACS 2018.2 were completed. Initial structures were derived from docking results. The workflow included topology generation, solvation, energy minimization, equilibration, and 30 ns production simulations. Analyses included RMSD, radius of gyration, solvent-accessible surface area (SASA), and hydrogen bond quantification [15, 16, 17].

## Free binding energy calculation:

Following 30 ns of MD simulations, free binding energy calculations were conducted for the various complexes using the g_mmpbsa package. This package integrates GROMACS and APBS, leveraging the MM-PBSA method to compute binding energy components, excluding the entropic term. It includes an energy decomposition scheme that evaluates the energetic contribution of each residue to the binding process. The output from these calculations serves as input for Python scripts within the package, enabling the determination of the final binding energy [18].

## Results & Discussion:

## Phytochemical library preparation and virtual screening:

A library of 545 phytochemicals, including their references, chemical names, and PubChem IDs, was extracted from the Moroccan Phytochemicals Database (MPDB) [11]. Virtual screening via PyRx identified 6-Hydroxygenistein (6-OHG), a derivative of Genistein, as a compound with high binding affinity to PDGFRα and c-KIT (Table 1 and Table 2). 6-OHG shows binding affinities of -10.3kcal/mol for PDGFRα and -10.5kcal/mol for c-KIT. Genistein displayed binding affinities of -9.6kcal/mol for PDGFRα and -9.8kcal/mol for c-KIT, providing a basis for comparative analysis.

## Docking results and interaction analysis:

**Figure 1:**
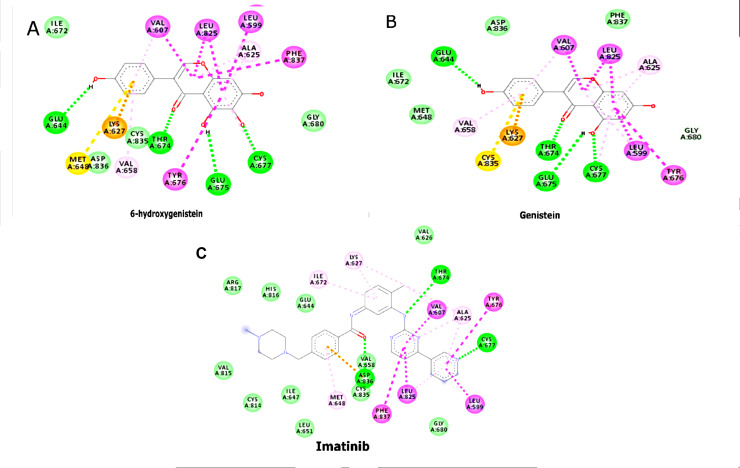
2D Interactions (A) 6-hydroxygenistein with PDGFRα residues; (B) Genistein with PDGFRα residues; (C) Imatinib with PDGFRα residues.

**Figure 2:**
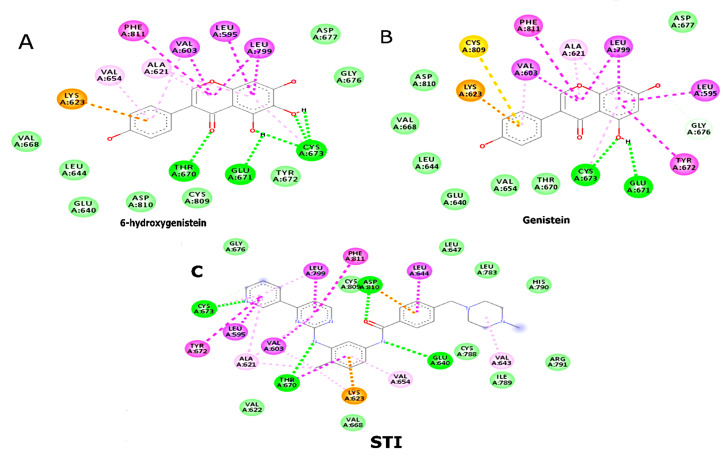
2D Interactions (A) 6-hydroxygenistein with c-KIT residues; (B) Genistein with c-KIT residues; (C) STI571 (Imatinib) with c-KIT residues.

Molecular docking studies have highlighted the stability and efficacy of 6-OHG and Genistein as potential dual inhibitors. Both compounds form significant interactions with PDGFRα and c-KIT. For PDGFRα, 6-OHG interacts via hydrogen bonds with residues such as Lys599 and Asp810 and several hydrophobic interactions that stabilize the ligand within the binding pocket, including Glu644, Thr674, Glu675, and Cys677, and exhibits Pi-Pi stacking with Phe837 and Tyr676 at the benzene ring structure in PDGFRα. Similarly, Genistein forms hydrogen bonds with the same critical residues and hydrophobic interactions, indicating its potential efficacy. These interactions are crucial for the ligand's stability and efficacy, closely mirroring the binding mechanisms of Imatinib (Figure 1). For c-KIT, 6-OHG also exhibited significant hydrogen bonding with residues such as Tyr823 and Asp810, alongside hydrophobic contacts in Thr670, Glu671, and Cys673. It also displayed Pi-Pi stacking with Phe811 at the benzene ring structure (Figure 2). The consistency in binding interactions across these phytochemicals underscores their potential as dual inhibitors.

## Drug-Like properties and ADMET prediction:

ADMET predictions (Table 3) confirmed that 6-OHG and Genistein possess favorable drug-like properties. 6-OHG has a molecular weight of 286.24 g/mol, a logP value of 2.06, and high solubility, all of which are within the acceptable range for drug development. Genistein also demonstrated favorable properties with a molecular weight of 270.24 g/mol and a log P value of 1.91, indicating good solubility and oral bioavailability [14]. These properties suggest that both compounds have the necessary pharmacokinetic attributes for further development as therapeutic agents.

## Molecular dynamics simulations:

**Figure 3:**
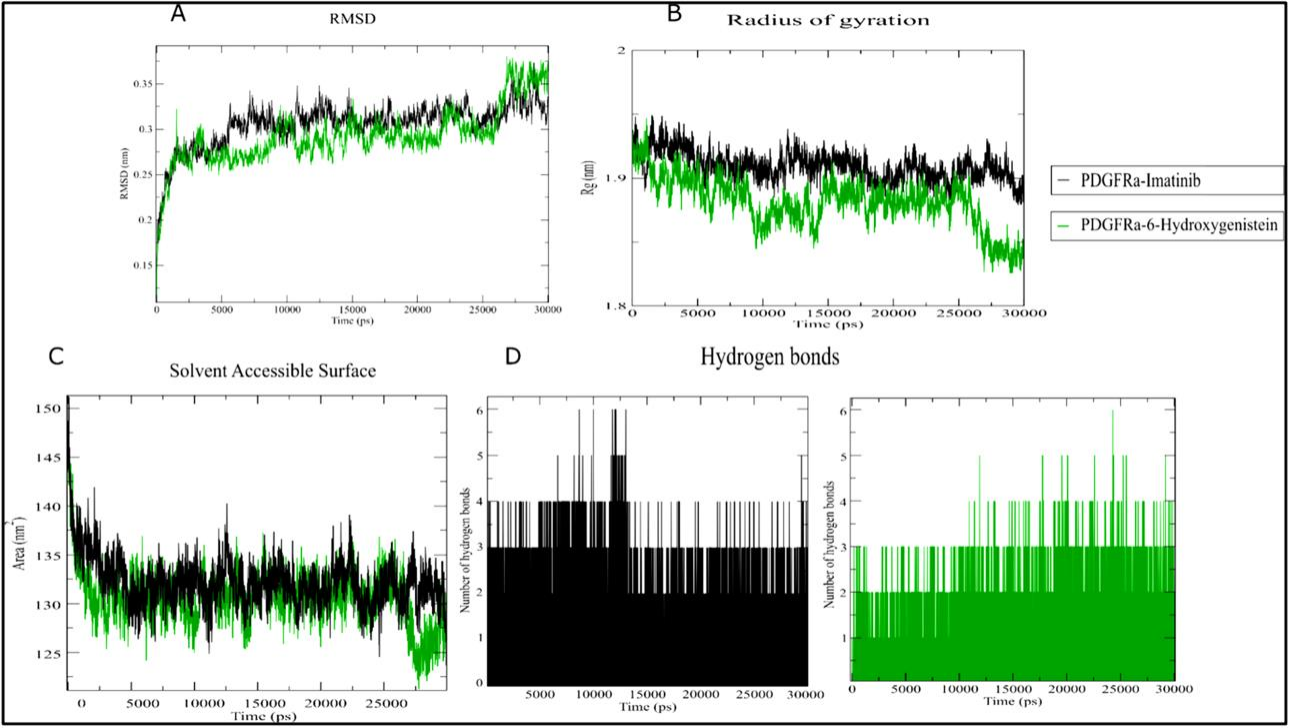
Molecular dynamics analysis of the PDGFRα/6-Hydroxygenistein-ligand complex during 30 ns simulations with Imatinib as a control. (A) RMSD, (B) Radius of gyration, (C) Solvent Accessible Surface, (D) Hydrogen bonds.

**Figure 4:**
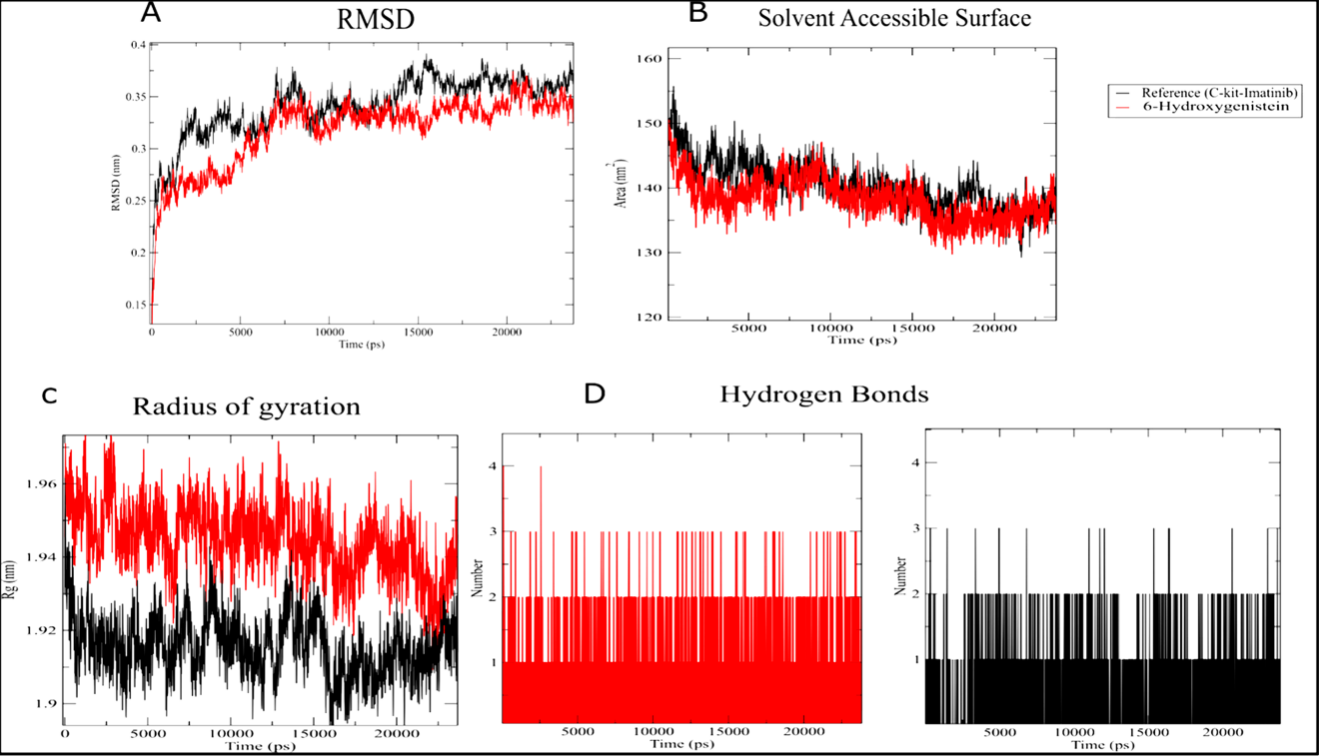
Molecular dynamics analysis of the c-KIT/6-Hydroxygenistein-ligand complex during 30 ns simulations with Imatinib as a control. (A) RMSD, (B) Radius of gyration, (C) Solvent Accessible Surface, (D) Hydrogen bonds.

Molecular dynamics (MD) simulations over a 30 ns period for the PDGFRα/6-OHG and c-KIT/6-OHG complexes provided insights into the stability and behavior of these interactions over time. The RMSD analysis indicated stable complexes with minor fluctuations (Figure 3). Notably, the RMSD trajectory of 6-OHG tended to stabilize after five ns, with a final RMSD value of approximately 2.0 Å, significantly lower than that observed for Imatinib (2.5 Å). The radius of gyration (Rg) remained consistent, reflecting the structural integrity of the complexes 6-OHG/PDGFRα and 6-OHG/c-KIT, with average values of 1.95 nm and 1.96 nm, respectively (Figure 4). The solvent-accessible surface area (SASA) showed stable values of 150.01 nm^2^ for 6-OHG/PDGFRα and 145.34 nm^2^ for 6-OHG/c-KIT complexes, suggesting consistent solvent exposure with minor conformational changes. The number of hydrogen bonds maintained throughout the simulation period was relatively stable. Notably, in the 6-OHG/PDGFRα complex, four hydrogen bonds were observed between the protein and drug. In comparison, the 6-OHG/c-KIT complex exhibited an average of 6 intermolecular hydrogen bonds, underscoring the ligand-protein interactions' robustness.

## Free binding energy calculations:

Free binding energy calculations using the MM-PBSA (Table 4) method highlighted the strong binding efficacy of 6-OHG and Genistein with PDGFRα and c-KIT. The total binding energy for 6-OHG was -278.0 kcal/mol with PDGFRα and -202.1 kcal/mol with c-KIT, surpassing the binding energies of Imatinib, which were -239.4 kcal/mol and -156.0 kcal/mol respectively [18].

## Antioxidant properties and toxicity profile:

Research indicates that 6-OHG has promising antioxidant properties, which could contribute to a potentially lower toxicity profile. The antioxidant activity of 6-OHG is similar to or even greater than that of vitamin C, suggesting its potential as a beneficial compound in reducing cell oxidative stress [19]. A study comparing the metabolism of Genistein and its derivatives in human and rat liver microsomes found that 6-OHG has a different metabolic profile, which could contribute to its distinctive biological effects and possibly lower toxicity [20]. While direct toxicity data specific to 6-OHG are limited, the existing studies imply that it may have a safer profile than Genistein due to its potent antioxidant capabilities and unique metabolic pathways [19, 20]. These findings underscore the need for further experimental validation of 6-OHG to confirm its dual inhibitory effects and to explore its clinical potential in overcoming resistance mechanisms in GIST therapy.

## Conclusion:

This study identified 6-Hydroxygenistein (6-OHG) as a promising dual inhibitor of PDGFRα and c-KIT through extensive virtual screening, molecular docking, ADMET predictions, and molecular dynamics simulations. The ADMET profile of 6-OHG, with its favorable molecular weight, solubility, and oral bioavailability and further supports its potential as a therapeutic agent. Notably, the antioxidant properties of 6-OHG, which are similar to or greater than those of vitamin C, suggest a lower toxicity profile than Genistein. Future studies should focus on detailed in vitro and in vivo experiments to fully elucidate the therapeutic potential and safety profile of 6-OHG, especially in overcoming resistance mechanisms in gastrointestinal stromal tumors (GIST) therapy. In summary, this study comprehensively evaluates 6-OHG, highlighting its potential as a novel dual inhibitor with promising therapeutic properties and a favorable safety profile.

## Data availability:

All data generated or analyzed during this study are included in this published article.

## Figures and Tables

**Table 1:** Binding affinities of moroccan plant-derived compounds to PDGFRα

**Ligand**	**Binding affinity to PDGFRα (kcal/mol)**
Imatinib	-13.1
6-Hydroxygenistein	-10.3
Schottenol	-10.7
Genistein	-9.6

**Table 2:** Binding affinities of moroccan plant-derived compounds to c-KIT

**Ligand**	**Binding Affinity to c-KIT (kcal/mol)**
Imatinib (STI)	-14.2
6-Hydroxygenistein	-10.5
Genistein	-9.8
Apigenin7-allosyl(1→2)glucoside	-9.7

**Table 3:** ADMET Profiles of Genistein and 6-Hydroxygenistein

**Compound**	**MW (g/mol)**	**logP**	**HBD**	**HBA**	**Solubility**	**Oral Bioavailability**	**Result**
Imatinib	493.6	4.04	2	6	Moderately soluble	Moderate	Accepted
Genistein	270.24	1.91	3	5	Good Solubility	Good	Accepted
6-Hydroxygenistein	286.24	2.06	4	6	Good Solubility	Good	Accepted

**Table 4:** Comparative Free Binding Energy of Imatinib and 6-Hydroxygenistein to PDGFRα and c-KIT using MMPBSA (Energies in kilocalories per mole)

**Complex**	**ΔEvdW**	**ΔEelec**	**ΔGpolar**	**ΔGnonpolar**	**ΔGbinding**
Imatinib - PDGFRα	-121.8 ± 14.6	-158.9 ± 26.7	46.0 ± 7.5	-4.7 ± 4.6	-239.4 ± 53.4
6-OHG - PDGFRα	-191.7 ± 10.7	-120.9 ± 14.6	48.1 ± 32.9	-14.8 ± 2.6	-278.0 ± 34.3
Imatinib - c-KIT	-185.6 ± 19.7	-59.3 ± 10.8	123.0 ± 5.2	-34.1 ± 2.6	-156.0 ± 38.5
6-OHG - c-KIT	-260.6 ± 10.7	-45.3 ± 9.8	123.0 ± 5.2	-19.1 ± 2.6	-202.1 ± 28.5
